# Mercury-Tolerant *Ensifer medicae* Strains Display High Mercuric Reductase Activity and a Protective Effect on Nitrogen Fixation in *Medicago truncatula* Nodules Under Mercury Stress

**DOI:** 10.3389/fpls.2020.560768

**Published:** 2021-01-14

**Authors:** Gabriela Arregui, Pablo Hipólito, Beatriz Pallol, Victoria Lara-Dampier, Diego García-Rodríguez, Higinio P. Varela, Parinaz Tavakoli Zaniani, Dimitrios Balomenos, Timothy Paape, Teodoro Coba de la Peña, M. Mercedes Lucas, José J. Pueyo

**Affiliations:** ^1^Department of Soil, Plant and Environmental Quality, Institute of Agricultural Sciences, ICA-CSIC, Madrid, Spain; ^2^Centro Nacional de Biotecnología, CNB-CSIC, Madrid, Spain; ^3^Brookhaven National Laboratory, Upton, NY, United States; ^4^Centro de Estudios Avanzados en Zonas Áridas, CEAZA, La Serena, Chile

**Keywords:** rhizobia, mercury, mercuric reductase, *merA*, nitrogen fixation, nodule, *Ensifer medicae*, *Medicago truncatula*

## Abstract

Mercury (Hg) is extremely toxic for all living organisms. Hg-tolerant symbiotic rhizobia have the potential to increase legume tolerance, and to our knowledge, the mechanisms underlying Hg tolerance in rhizobia have not been investigated to date. Rhizobial strains of *Ensifer medicae*, *Rhizobium leguminosarum* bv. *trifolii* and *Bradyrhizobium canariense* previously isolated from severely Hg-contaminated soils showed different levels of Hg tolerance. The ability of the strains to reduce mercury Hg^2+^ to Hg^0^, a volatile and less toxic form of mercury, was assessed using a Hg volatilization assay. In general, tolerant strains displayed high mercuric reductase activity, which appeared to be inducible in some strains when grown at a sub-lethal HgCl_2_ concentration. A strong correlation between Hg tolerance and mercuric reductase activity was observed for *E. medicae* strains, whereas this was not the case for the *B. canariense* strains, suggesting that additional Hg tolerance mechanisms could be playing a role in *B. canariense*. Transcript abundance from *merA*, the gene that encodes mercuric reductase, was quantified in tolerant and sensitive *E. medicae* and *R. leguminosarum* strains. Tolerant strains presented higher *merA* expression than sensitive ones, and an increase in transcript abundance was observed for some strains when bacteria were grown in the presence of a sub-lethal HgCl_2_ concentration. These results suggest a regulation of mercuric reductase in rhizobia. Expression of *merA* genes and mercuric reductase activity were confirmed in *Medicago truncatula* nodules formed by a sensitive or a tolerant *E. medicae* strain. Transcript accumulation in nodules formed by the tolerant strain increased when Hg stress was applied, while a significant decrease in expression occurred upon stress application in nodules formed by the Hg-sensitive strain. The effect of Hg stress on nitrogen fixation was evaluated, and in our experimental conditions, nitrogenase activity was not affected in nodules formed by the tolerant strain, while a significant decrease in activity was observed in nodules elicited by the Hg-sensitive bacteria. Our results suggest that the combination of tolerant legumes with tolerant rhizobia constitutes a potentially powerful tool in the bioremediation of Hg-contaminated soils.

## Introduction

Mercury is the most toxic heavy metal, and mercury contamination is becoming a major problem worldwide, in both wild ecosystems and agricultural soils. Environmental mercury contamination can be due to natural processes (volcanism, soil erosion, geothermal activities, and wild fires), but is mostly the result of anthropogenic activities, such as mining or the increasing use of phytochemicals in agriculture (Dash and Das, 2012; Wu et al., 2014; Paris et al., 2015; Marx et al., 2016). The most prevalent forms of mercury in the environment and in living organisms are metallic mercury (Hg^0^), mercuric mercury (Hg^2+^), and methylmercury (CH_3_Hg^+^), which is formed as a result of the methylation of Hg^2+^ by some microorganisms found in soil and water (Dash and Das, 2012; Tchounwou et al., 2012; Naguib et al., 2018). All forms of Hg are toxic, but Hg^0^ is volatile and the least toxic mercury state (Barkay et al., 2003). The toxicity of Hg and other heavy metals is due to complex interactions with cellular macromolecules (Azevedo and Rodriguez, 2012). In bacteria, plants and other organisms, the presence of metals might cause the formation of free radicals, generating oxidative stress (Mudipalli, 2008), and replacement of essential metals in pigments or enzymes, thus affecting their optimal activity (Malayeri et al., 2008; Mathema et al., 2011). Mercury appears to enter in both prokaryotic and eukaryotic cells through ion channels, competing with essential metals such as copper, iron, or zinc (Blazka and Shaikh, 1992; Bridges and Zalups, 2005). It has a high affinity for the sulfhydryl groups of amino acids, causing the alteration and loss of function of enzymes and sulfur-containing antioxidants (Patra and Sharma, 2000; Nies, 2003). Consequently, high bioaccumulation of mercury presents considerable problems in plants and animals, and results in prominent biomagnification in the trophic chain (David et al., 2012; Lavoie et al., 2013).

Some microorganisms have developed different strategies to protect themselves against mercury, such as extrusion by ATPases, cation antiport systems, immobilization with anionic ligands or precipitation of colloids (Robinson and Tuovinen, 1984; Osborn et al., 1997; Naguib et al., 2018). A common strategy to many bacteria is the enzymatic reduction of Hg^2+^ to less toxic and volatile Hg^0^, which is mediated by the bacterial mercuric reductase enzyme (MerA) encoded by the *merA* gene, which is usually part of the *mer* operon that contains several genes involved in mercury detoxification. Operon components and organization may be different in different bacteria (Mathema et al., 2011; Boyd and Barkay, 2012; Freedman et al., 2012; Naguib et al., 2018).

Mercury-tolerant microorganisms capable of detoxifying mercury have a potential for bioremediation of mercury-contaminated water and soil (Barkay et al., 2003). Therefore, the identification of tolerant, mercury-detoxifying bacteria is highly relevant. Deficiencies in nutrients, such as nitrogen, are the main limiting factors in the revegetation and/or phytoremediation of poor, marginal and polluted soils. Rhizobia are gram-negative soil bacteria that fix atmospheric nitrogen in the symbiotic root nodules that they form with leguminous plants. Once bacteria are released inside the host plant cell, they differentiate into bacteroids, the nitrogen-fixing form. Both host plant and rhizobia benefit from this interaction because the plant obtains nitrogen as ammonia (NH_3_), which is rapidly protonated to ammonium (NH_4_^+^), while the bacteria receive carbon substrates (succinate and malate) as a way to rapidly obtain energy (Udvardi and Poole, 2013). This is why the use of rhizobia-legume symbioses has been proposed to be a useful tool for the bioremediation of nutrient-depleted heavy metal-contaminated soils (Hao et al., 2014). The symbiotic system has actually been successfully used in soils contaminated with different heavy metals (Dary et al., 2010; Fagorzi et al., 2018; Bellabarba et al., 2019). Thus, the identification of Hg-tolerant rhizobia strains capable of detoxifying mercury has become of great interest to be used in conjunction with tolerant legumes in Hg-polluted soils.

One of the largest deposits of mercury in the world is the Almadén mine district in Central Spain, where the primary mineral is cinnabar (mercury sulfide). Centuries of mining caused the dispersion of the metal in the environment surrounding Almadén, leading to elevated mercury concentrations in soils and plants (Martínez-Coronado et al., 2011; Lominchar et al., 2015). To our knowledge, the tolerance and detoxification mechanisms present in Hg-resistant rhizobia have not yet been described. The aim of this study was to analyze rhizobial strains collected from Hg-contaminated soils of Almadén, which presented different tolerance to Hg, in order to identify whether mercury tolerance was due to their capacity to reduce Hg^2+^ to Hg^0^. Bacteria strains of *Ensifer* (formerly *Sinorhizobium*) *medicae*, *Rhizobium leguminosarum* bv. *trifolii*, and *Bradyrhizobium canariense* were previously isolated from nodules in host plants growing in Hg-contaminated soils (Nonnoi et al., 2012; Ruiz-Díez et al., 2012, 2013). We hypothesize that significant differences in mercury reductase activity will be detected between strains having low vs. high mercury tolerance in free living conditions and that the enzyme might be active and provide a protective effect on nitrogen fixation in legume nodules in the presence of Hg stress.

## Materials and Methods

### Bacteria and Growth Media

Bacteria used in this study were previously isolated from legume nodules that were grown in Hg-contaminated soil (see Nonnoi et al., 2012; Ruiz-Díez et al., 2012, 2013 for specific information regarding each strain). Twenty-one *R. leguminosarum* bv. *trifolii* strains and 23 *Ensifer medicae* strains were isolated from nodules of *Trifolium* and *Medicago* species, respectively, which were growing in different sites near the abandoned Almadén mercury mine in Spain. Seven *B. canariense* strains were isolated from nodules of *Lupinus, Spartium, Adenocarpus*, and *Ornithopus* species collected from the Almadén area or from non-contaminated sites. *E. medicae* and *R. leguminosarum* strains were grown in Tryptone Yeast (TY) medium, and *B. canariense* strains were grown in Vincent medium (Vincent, 1970).

### Rhizobial Tolerance to Mercury: Minimum Inhibitory Concentration

In all experiments described in this work, HgCl_2_ was used as the source of Hg. Hereafter, Hg, Hg tolerance, Hg-tolerant, Hg-sensitive, Hg stress, Hg concentration always refers to HgCl_2_. Rhizobial strains were grown in liquid medium at 28°C until an OD_600_ of 0.7. To determine the lowest HgCl_2_ concentration that completely inhibits bacterial growth, minimum inhibitory concentration (MIC), 2 μl of bacterial culture was inoculated on agar solid medium with increasing HgCl_2_ concentrations: 25, 50, 75, 100, 125, 150, 175, 200, 225, 250, and 275 μM (Nonnoi et al., 2012). The inoculated plates were incubated at 28°C. *E. medicae* and *R. leguminosarum* are fast-growing rhizobia that, in our experimental conditions, showed visible growth after 2 d when grown in the absence of HgCl_2_. *B. canariense* is a slow-growing rhizobium and growth was not evident before 7 d. Thus, MIC was determined after 2 d for *E. medicae* and *R. leguminosarum*, and after 7 d for *B. canariense*.

### Mercury Volatilization Assay

The ability of rhizobia to reduce Hg^2+^ to Hg^0^ was analyzed by an X-ray film method that detects mercury volatilization. When Hg^0^ is generated, mercury vapor reacts with silver (Ag^+^) contained in the X-ray film, it shows a dark spot (Nakamura and Nakahara, 1988). Rhizobia strains were grown in liquid medium in the absence of HgCl_2_ or a concentration of 4 μM HgCl_2_ in order to determine an induction of mercuric reductase activity by mercury. Cultures were grown at 28°C until the concentration reached an OD_600_ of 0.7. Then, 4 ml of each culture were centrifuged at 2,000 × g for 15 min at 20°C. The pellet was resuspended in 50 μl of reaction medium that contained 50 μM HgCl_2_ (Nakamura and Nakahara, 1988). Controls contained the reaction medium without bacteria. Reaction mixtures were loaded onto a multi-well plate, and an X-ray film was placed on the plate in the dark (Nakamura and Nakahara, 1988). Films were developed after 4 h. Reaction medium without bacteria was used as a negative control. A densitometry analysis of the X-ray films was performed using the ImageJ software.

### Transcriptional Analyses of Mercuric Reductases

*E. medicae* strains AMp08 (tolerant) and VMo01 (sensitive) and *R. leguminosarum* strains STf07 (tolerant) and VTc11 (sensitive) were grown in liquid TY medium in the absence or presence of mercury (4 μM HgCl_2_). When cultures reached an OD_600_ of 0.8, we directly added the Qiagen RNAprotect Bacteria Reagent to the bacterial culture in a 1:2 ratio (culture: RNA stabilizer). The mixtures were vortexed for 5 s and incubated for 5 min at room temperature (23°C) and then centrifuged at 5,000 × *g* for 10 min at 20°C. Pellets were frozen in liquid nitrogen. Bacterial lysis was performed by resuspension and incubation of the cell pellet in 1 mg/ml lysozyme from chicken egg whites (Sigma-Aldrich) in Tris–EDTA buffer, pH 8.0. Total RNA was extracted using the Qiagen RNeasy Mini Kit. For gene expression in bacteroids, nodules of *M. truncatula* were frozen in liquid nitrogen and the tissue was ground with using the MM40 (Retsch) mixer mill. RNA was extracted using the Qiagen RNeasy Plant Mini Kit according to the manufacturer’s protocol. The isolated RNA was subjected to DNase treatment. The quality and the quantity of the extracts were firstly analyzed with a NanoDrop 1,000 spectrophotometer (Thermo Scientific), and subsequently, RNA quality was assessed with an Agilent 2,100 Bioanalyzer.

For quantitative real time PCR (qPCR), reverse transcription was performed using the High-Capacity cDNA Reverse Transcription Kit (Applied Biosystems). Reactions were performed in a 7300 Real Time PCR System (Applied Biosystem). Each qPCR reaction contained 7.5 μl of SYBR Green PCR master mix (PE Applied Biosystem), 5 μl of cDNA, and 10 ng/μl of each primer in a final volume of 15 μl. Minus-reverse transcriptase (–RT) controls of the RNA samples were included in real-time RT-PCR experiments, in order to detect and estimate genomic DNA contamination; only samples free of genomic DNA contamination were included. Primers were designed using Primer Express Software v3.0 (PE Applied Biosystems). Primers used to amplify *E. medicae merA1* and *merA2* genes and *R. leguminosarum merA2* are listed in Table 1. Genes encoding 16S rRNA genes were used as endogenous controls. Thermocycling conditions were as follows: an initial denaturing time of 10 min at 95°C, followed by 40 PCR cycles consisting of 95°C for 15 s and 60°C for 1 min. The data were analyzed using the 7,300 System Software (Applied Biosystems). And the relative quantification was carried out according to the comparative C_T_ method (Pfaffl, 2001), as described in the Applied Biosystems StepOne^TM^ Real-Time PCR System. Three independent biological experiments were performed with three technical replicates per sample. Each nodule sample contained nodules pooled from 4–6 plants. Differences were considered significant when the fold-change was ≥2 and SD bars did not overlap.

**TABLE 1:** Primers used for qPCR amplification of *merA* genes.

Species	Primer	Sequence (5*′ –* 3*′*)	Reference strains and genes
*E. medicae*	f-merA1.Emed	TCGCCGTTGCCAATCG	*E. medicae* WSM419
	r-merA1.Emed	CGGATAGGGTGCGACATAAGTC	Acc. N. NC_009636 (Smed_0084)
	f-merA2.Emed	CGCATCGACGATCACTTTCA	*E. medicae* WSM419
	r-merA2.Emed	CCGCGCGGTCGAAAA	Acc. N. NC_009620 (Smed_4636)
	f-16SrRNA.E.med	CCTTACGGGCTGGGCTAC	*E. medicae* WSM419
	r-16SrRNA.E.med	GGTCTCGCTGCCCACTGT	Acc. N. CP000738 (Smed_R0041)
*R. leguminosarum* bv. *trifolii*	f-merA2.Rleg	GCGATGAATGCAGGAACGA	*R. leguminosarum* bv. *trifolii* WSM1325
	r-merA2.Rleg	GACACCGTCGGATGGATAGG	Acc. No. NC_012858 (Rleg_6744)
	f-16SrRNA.Rleg	CCGACGGCTAACATTCATCG	*R. leguminosarum* bv. *trifolii* WSM1325
	r-16SrRNA.Rleg	CATTACTGACGCTGAGGTGC	Acc. No. CP0011622 (Rleg_R0040)

*Reference strains and genes used for primer design are indicated.*

### Phylogenetic Analysis

Alignment and phylogenetic analysis of mercuric reductase (MerA) protein sequences of selected rhizobia were performed using the Geneious 6.01 software (Kearse et al., 2012). The neighbor-joining method (Saitou and Nei, 1987) was applied to construct a phylogenetic tree. The evolutionary distances were computed with the Jukes-Cantor genetic distance amino acid substitution model. Branch support was generated using bootstrapping of 100 resampled trees.

### Plant Material, Inoculation, Growth Conditions, and Mercury Stress

*Medicago truncatula* cv. Parabinga plants were used in this study. *M. truncatula* seeds were scarified with 96% sulfuric acid for 6 min, and then immersed in commercial bleach for 1 min, washed 6 times with sterile distilled water and left in water for 1 h. Sterilized seeds germinated overnight on Petri dishes containing 1% agar/water. Germinated seeds were inoculated during sowing with 1 ml of bacteria grown to exponential phase (OD_600_ = 0.8). Two *E. medicae* strains were used: AMp08, the strain that displayed the highest mercury tolerance, and VMo01, a sensitive strain that showed an increase in mercuric reductase activity when grown at a sub-lethal mercury concentration (4 μM HgCl_2_).

Plants were grown in pots filled with vermiculite in a growth chamber (25/19°C, 16/8 h photoperiod) and irrigated with nitrogen-free nutrient solution (Hoagland and Arnon, 1938). After 5 weeks, plants were watered with nutrient solution containing 0 mM or 500 μM HgCl_2_. Plant material was collected 24 h after treatment application.

### Nitrogenase Activity

Nitrogen fixation activity was estimated by the acetylene reduction assay (ARA), as described by Shvaleva et al. (2010). Although the use of a “closed” system for measuring acetylene reduction can lead to an acetylene-induced decline in nitrogenase activity, it is appropriate for comparative measurements, especially when assay time is short. Nodulated roots were introduced in 15.5 ml tubes and 10% of volume was replaced by acetylene. After 1 h of incubation, gas samples were taken and analyzed for ethylene content using a gas chromatographer (Perkin–Elmer 8310, United States) using nitrogen as a carrier gas. We took into account the volume of the nodulated roots after measurements were taken; nodules were detached and weighed to calculate the nitrogenase activity per gram of nodule. Three independent biological experiments were performed. In each experiment, 7–10 plants per treatment were analyzed. Statistics analysis were performed with IBM SPSS Statistics 25 (SPSS Inc., Chicago, IL, United States), using a mixed linear model to analyze the effect of Hg on the nitrogenase activity (*p* < 0.05).

## Results

### Mercury Tolerance and Mercuric Reductase Activity in Free-Living Rhizobia

Mercury tolerance of rhizobia strains was tested on solid medium by measuring MIC following treatment with Hg (Table 2). Four *E. medicae* strains (AMp08, AMp10, NMp01, y SMp01) and three *R. leguminosarum* strains (STf07, STf08, and STf09) are considered Hg-tolerant (Nieto et al., 1989) as their growth was not inhibited in 0.1 mM Hg (MIC > 100 μM). All *B. canariense* strains were sensitive to mercury. L7-AH was the most Hg-tolerant *B. canariense* strain (MIC = 50 μM), while ISLU-16 and L-3, which were obtained from non-contaminated sites, were the most Hg-sensitive strains. The MIC values obtained for *B. canariense* in solid medium were higher than those previously reported, which had been measured in liquid medium (Ruiz-Díez et al., 2012, 2013). Some possible reasons that might explain the observed differences are considered below in the Discussion section.

**TABLE 2:** Rhizobial strains used in this study.

Strain	Bacterial species	Host plant species	MIC (μM)	MerA activity (0 μM HgCl_2_) (densitogram peak area) Mean ± SD	MerA activity (4 μM HgCl_2_) (densitogram peak area) Mean ± SD
AMp01	*Ensifer medicae*	*Medicago polimorpha*	50	11.70 ± 3.88	18.95 ± 2.32
AMp02			50	21.43 ± 5.02	32.58 ± 2.28
AMp04			75	23.03 ± 2.93	35.86 ± 4.03
AMp07			75	31.74 ± 1.43	46.32 ± 1.98
AMp08			250	200.20 ± 24.51	233.14 ± 7.78
AMp09			100	75.65 ± 5.20	77.86 ± 3.89
AMp10			150	54.82 ± 3.94	103.49 ± 5.98
NMp01			150	77.86 ± 3.86	146.22 ± 7.90
NMp03			75	31.80 ± 3.82	47.10 ± 4.11
NMp04			50	22.26 ± 6.00	27.88 ± 2.90
NMp05			50	21.89 ± 3.03	56.10 ± 2.62
NMp06			50	19.53 ± 3.36	30.19 ± 1.89
NMp07			50	15.44 ± 0.59	32.67 ± 5.21
NMp08			50	23.82 ± 0.34	14.67 ± 2.47
NMp09			50	29.30 ± 0.85	30.21 ± 1.70
NMp10			50	34.47 ± 2.07	44.27 ± 2.81
NMp11			50	27.04 ± 0.61	36.62 ± 3.51
NMp12			50	25.95 ± 2.77	26.02 ± 1.26
NMp13			50	39.39 ± 3.25	62.18 ± 2.27
SMp01		*Medicago orbicularis*	200	221.23 ± 7.14	265.83 ± 16.39
VMo01			50	50.35 ± 1.84	98.96 ± 6.64
VMo03			50	16.76 ± 3.23	21.88 ± 7.16
VMo04			50	18.56 ± 0.46	33.95 ± 10.14
STf06	*Rhizobium Leguminosarum* bv. *trifolii*	*Trifolium fragiferum*	25	18.07 ± 3.94	28.32 ± 0.73
STf07			250	223.64 ± 9.76	228.48 ± 7.12
STf08			225	58.50 ± 4.47	218.05 ± 11.58
STf09			175	270.32 ± 4.76	253.11 ± 10.86
VTc07		*Trifolium campestre*	25	17.95 ± 3.24	34.73 ± 2.78
VTc08			25	24.61 ± 2.94	28.43 ± 3.12
VTc09			25	30.47 ± 0.15	17.96 ± 1.64
VTc10			25	28.10 ± 2.98	11.09 ± 1.94
VTc11			25	10.18 ± 2.24	33.58 ± 4.36
VTg12		*Trifolium glomeratum*	25	12.10 ± 2.47	7.67 ± 1.87
VTg13			25	6.65 ± 1.53	8.32 ± 0.68
VTg14			25	26.66 ± 2.60	44.76 ± 1.19
VTs15		*Trifolium* sp.	25	40.72 ± 3.71	35.88 ± 5.18
VTs16			25	17.28 ± 1.66	13.73 ± 0.90
VTs17			25	15.74 ± 0.72	29.82 ± 1.89
VTs18			25	11.13 ± 0.73	8.24 ± 1.66
VTs19			25	30.15 ± 5.41	15.64 ± 0.66
VTs20			25	14.28 ± 0.71	30.79 ± 0.75
VTs21			25	16.53 ± 1.06	23.13 ± 1.00
VTs22			25	10.94 ± 2.26	22.31 ± 3.37
VTs23			25	7.35 ± 1.90	20.70 ± 1.20
A-7C	*Bradyrhizobium canariense*	*Adenocarpus hispanicus*	15	78.38 ± 23.60	214.04 ± 15.21
ISLU-16		*Ornithopus compressus*	6	10.29 ± 2.51	no growth
L-3		*Lupinus albus*	6	11.64 ± 0.93	no growth
L7-AH			50	142.20 ± 11.37	165.94 ± 10.95
L-7Q			15	9.23 ± 0.89	no growth
SP-7C1		*Spartium junceum*	25	166.05 ± 10.34	309.98 ± 17.51
SP-7C2			25	50.86 ± 5.88	87.41 ± 3.10

*Bacterial species, host plant species, MIC and MerA activity of the strains grown in the absence or presence of 4 μM HgCl_2_ are indicated.*

The X-ray film assays revealed the ability of the strains to reduce Hg^2+^ to more volatile Hg^0^. Mercuric reductase activity values were obtained from densitometry analyses of the X-ray films. In general *E. medicae* and *R. leguminosarum* tolerant strains showed high mercuric reductase activities. Additionally, many of the strains showed increased mercuric reductase activity when they were grown in the presence of a low mercury concentration. The mercuric reductase activity assays performed with *B. canariense* strains showed that some strains displayed mercuric reductase activity. In some cases, activity increased when the strains had been cultivated in the presence of 4 μM HgCl_2_ (Table 2). Strain SP-7C1, which presented the highest mercury reductase activity, was not the most tolerant strain. Strains ISLU-16, L-3, and L-7Q displayed very low reductase activity. Moreover, their growth in liquid medium was completely inhibited by 4 μM Hg, in agreement with the MIC values previously reported (Ruiz-Díez et al., 2012, 2013).

**FIGURE 1:**
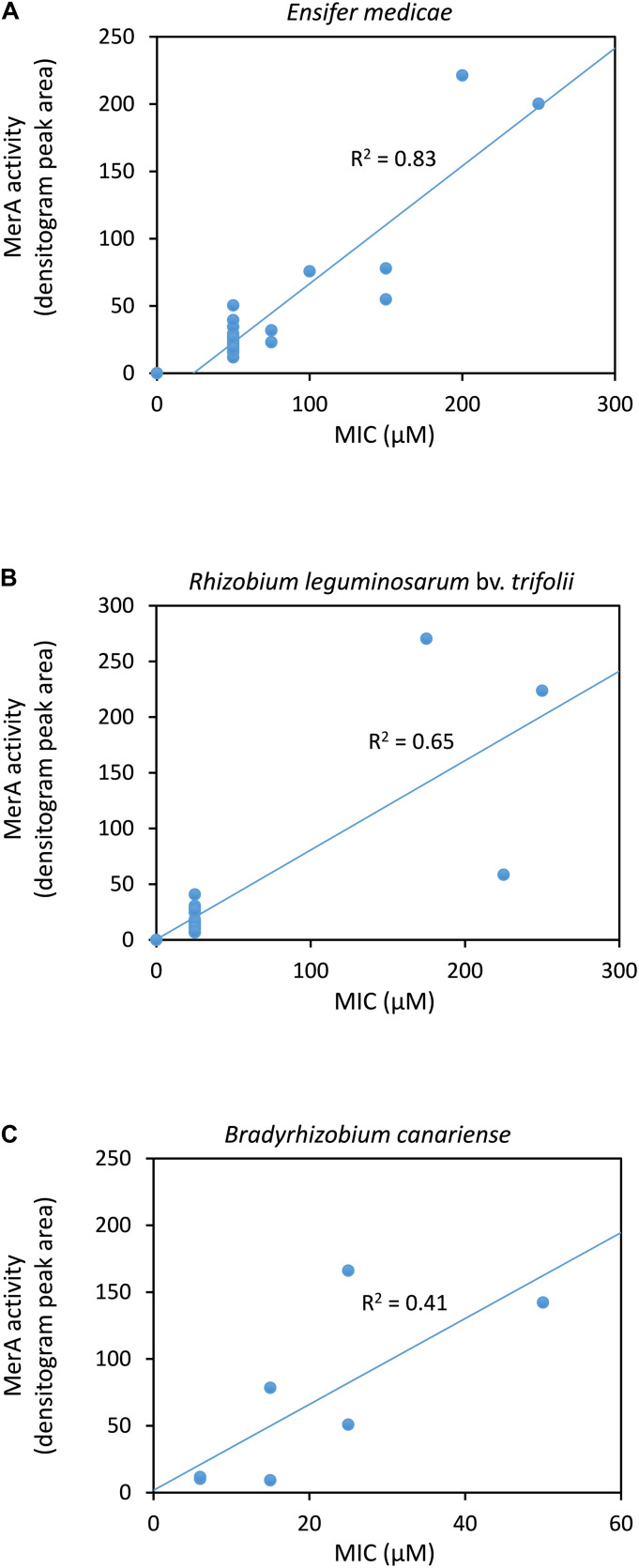
Correlation between mercuric reductase activity and mercury tolerance of rhizobial strains. **(A)** *Ensifer medicae*. **(B)** *Rhizobium leguminosarum* bv. *trifolii.* **(C)** *Bradyrhizobium canariense*. Mercuric reductase values were obtained from the densitometry analyses of the X-ray films. The coefficients of determination *R*^2^ are indicated.

In order to estimate the association of mercury tolerance of the rhizobial strains with their mercuric reductase activity, we performed a densitometry analysis of the rows in the X-ray films corresponding to the bacteria grown without mercury (rows 1 and 3). The densitometry values were plotted against the MIC values for *E. medicae* (Figure 1A), *R. leguminosarum* bv. *trifolii* (Figure 1B) and *B. canariense* (Figure 1C) strains. A strong correlation (*R*^2^ = 0.83) was observed in the case of *E. medicae*, while no correlation was found for the *B. canariense* strains (*R*^2^ = 0.41). We considered that the *R*^2^ value for *R. leguminosarum* might not be reliable, as there were only three strains that presented some degree of tolerance. It should also be noted that the X-ray film assay is a semi-quantitative assay, and consequently, the activity values obtained by densitometry analysis should be considered to be approximations.

### Expression of *merA* Genes in Free-Living Rhizobia

**FIGURE 2:**
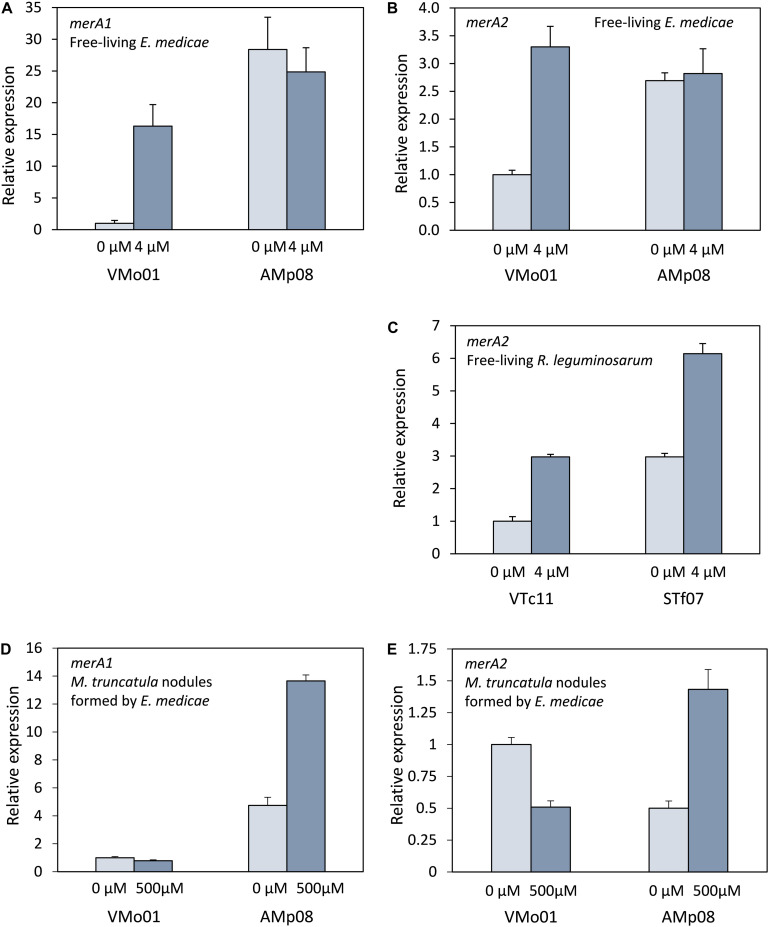
Expression of *merA* in free-living rhizobia and in *Medicago truncatula* nodules. **(A)** *merA1* expression in free-living *E. medicae* strains VMo01 (sensitive) and AMp08 (tolerant). **(B)** *merA2* expression in free-living *E. medicae* strains VMo01 and AMp08. **(C)** *merA2* expression in free-living *R. leguminosarum* bv. homologs were identified in some species *trifolii* strains VTc11 (sensitive) and STf07 (tolerant). **(D)** *merA1* expression in *M. truncatula* nodules formed by *E. medicae* strains VMo01 or AMp08. **(E)** *merA2* expression in *M. truncatula* nodules formed by *E. medicae* strains VMo01 or AMp08. Free-living bacteria were grown in the absence (0 μM) or presence of 4 μM HgCl_2_. Three independent biological experiments were performed. Five week-old nodulated *M. truncatula* plants were watered with nutrient solution containing 0 or 500 μM HgCl_2_ and harvested after 24 h. Three independent biological experiments were performed. Each biological sample contained nodules pooled from 4–6 plants. Three technical replicates per sample were performed. Mean values ± SD are represented. Differences were considered significant when fold change was ≥2 and SD bars did not overlap.

Our assays indicated that mercuric reductase activity was induced in some strains when grown in sublethal mercury concentrations (4 μM HgCl_2_). Two *E. medicae* strains, VMo01 (sensitive) and Amp08 (tolerant), and two *R. leguminosarum* strains, VTc11 (sensitive), and STf07 (tolerant) were used to determine whether the differences in activity between strains and the mercury-induced increase in activity observed corresponded with *merA* gene expression levels. Sequenced *E. medicae* WSM419 strain has two mercuric reductase genes, one located on the chromosome (*merA1*) and another one on the symbiotic plasmid pSymB (*merA2*). *R. leguminosarum* WSM1325 has only one mercuric reductase gene (*merA2*) located on plasmid pR132502. To compare the expression levels of the *merA* genes in the different strains grown in the absence or presence of a sub-lethal mercury concentration, qPCR was performed. In *E. medicae*, *merA1* expression was higher in the tolerant strain (AMp08) than in the sensitive strain (VMo01) when grown in the absence of HgCl_2_ (Figure 2A). The tolerant strain did not show a significant increase in expression when grown in the presence of HgCl_2_. In contrast, the expression of the *merA1* gene significantly increased when the sensitive strain was cultivated in the presence of HgCl_2_. It appeared that induction ocurred due to the presence of the metal in the growth medium. *E. medicae merA2* expression patterns were similar to those of *merA1* (Figure 2B). In the absence of Hg, expression was higher in the tolerant strain (AMp08) and a significant induction occurred when the sensitive strain (VMo01) was grown in the presence of mercury. Expression of *R. leguminosarum merA2* gene was higher in the tolerant strain (STf07) than in the sensitive strain (VTc11). There was an increase in transcript accumulation in both strains when they were grown in the presence of mercury (Figure 2C).

### *merA* Genes Expression, Mercuric Reductase Activity and Nitrogen Fixation in *M. truncatula* Nodules

The symbiotic interaction of rhizobia with legume roots leads to the formation of a new plant organ, the root nodule. Inside the nodule, bacteria differentiate into bacteroids, which are able to fix atmospheric nitrogen. In this process, some bacterial genes are inactivated, while others are overexpressed. Inactivation of the *merA* genes in bacteroids would imply the absence of mercuric reductase in the nodule and thus, the loss of an important mercury detoxification mechanism that could increase the plant tolerance to the metal. *M. truncatula* plants were inoculated with either *E. medicae* VMo01 (sensitive) or Amp08 (tolerant) strains, and after 5 weeks, when nodules are mature and active in nitrogen fixation, mercury stress was applied. We performed qPCR to check whether *merA1* and *merA2* were expressed in bacteroids, and to detect any differences in expression between the rhizobial strains or the mercury treatment. Both *merA* genes were expressed in the nodule. Similar to what we had observed for free living bacteria, *merA1* expression (Figure 2D) was higher in the tolerant strain than in the sensitive one. In contrast, the tolerant strain showed an induction when mercury stress was applied, whereas the sensitive strain did not show any induction. In control (untreated with Hg) nodules, *merA2* expression was somewhat higher in the sensitive strain. While mercury stress led to a decrease in *merA2* expression in the sensitive strain, the tolerant strain showed an induction of gene expression (Figure 2E). Although the conditions were not comparable, these results contrast with the expression in free-living bacteria, where the tolerant strain showed a higher expression but there was not an induction, whereas the sensitive strain showed an induction when grown in the presence of HgCl_2_. The *merA* transcripts in the bacteroids were translated into active mercuric reductase, as activity could be detected in crushed nodules (data not shown). However, the mercuric reductase assay was not suitable to quantify differences in activity between strains, or between control and mercury-stressed nodules. The assay results were not reproducible most likely due to heterogeneity of the crushed nodule tissue.

**FIGURE 3:**
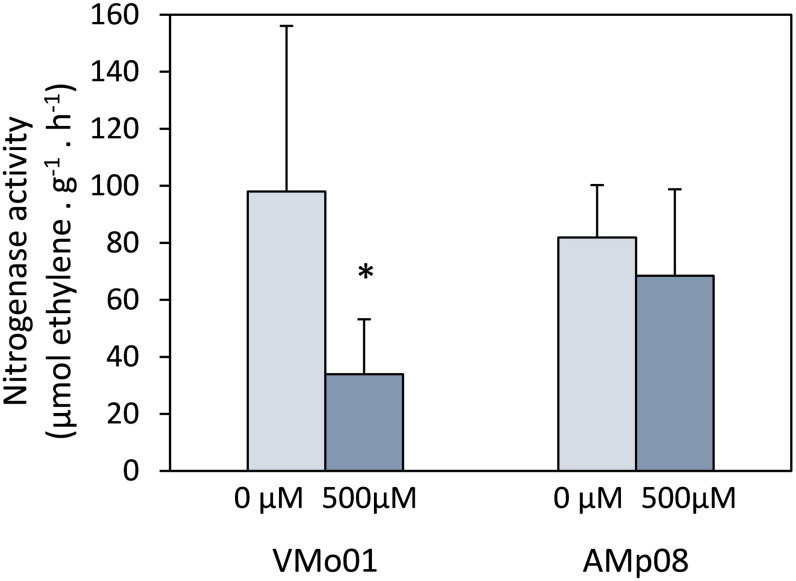
Nitrogenase activity per gram of nodule in control and Hg-treated *M. truncatula* nodules inoculated with *E. medicae* strains VMo01 (sensitive) and AMp08 (tolerant). Five week-old plants were watered with nutrient solution containing 0 or 500 μM HgCl_2_ and harvested after 24 h. Three independent biological experiments were performed. In each experiment, 7–10 plants per treatment were analyzed. Mean values ± SD are represented. An asterisk indicates significant differences (*p* < 0.05) between control and Hg-treated nodules.

Nitrogenase activity was analyzed in nodules of *M. truncatula* plants inoculated with *E. medicae* strains VMo01 (sensitive) or AMp08 (tolerant) (Figure 3). The symbiotic performance of plants inoculated with the sensitive or the tolerant strains was similar, as there were no significant differences in nitrogenase activity in non-stressed plants. Additionally, no significant differences were observed in root and shoot fresh weight due to the inoculated strain (data not shown). Nodules formed by the sensitive strain showed a significant decrease in nitrogenase activity (66%) when mercury stress was applied compared to control nodules, while no significant differences were observed for plants inoculated with the tolerant strain.

**FIGURE 4:**
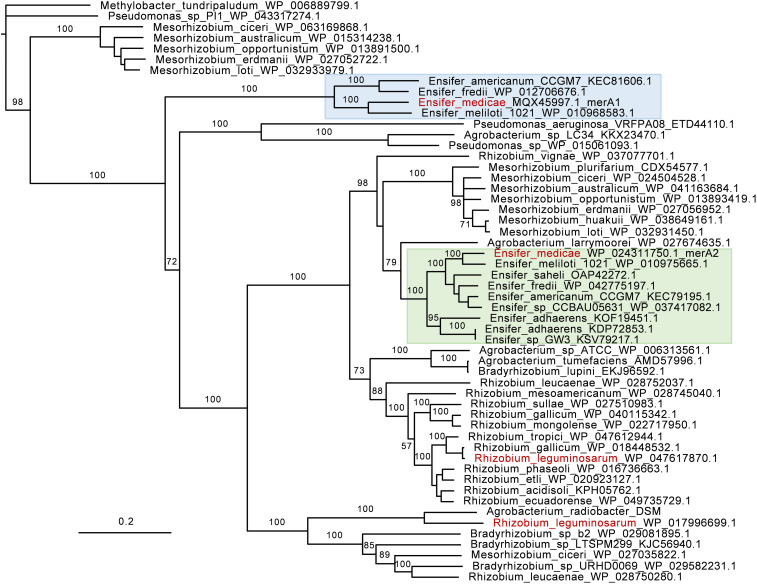
Phylogeny of mercuric reductase (MerA) in rhizobia. GenBank accession numbers are indicated. The phylogeny is based on amino acid sequences. The tree was rooted using two non-rhizobia MerA from *Methlybacter tundripaludum* and *Pseudomonas* sp. as outgroup sequences. The *Ensifer* sp. clades for MerA1 and MerA2 homologes are highlighted in blue and green, respectively. *Ensifer medicae* and *Rhizobium leguminosarum* are shown in red text.

To examine the prevalence and conservation of the *merA* genes among sequenced rhizobia, we performed a phylogenetic analysis. Widespread conservation of *merA* genes across rhizobia species was observed, as it was present in all species examined, including species from the genera *Agrobacterium, Bradyrhizobium, Mesorhizobium, Rhizobium*, and *Ensifer* (Figure 4). Two MerA homologs were identified in some species of the genera *Ensifer* and *Mesorhizobium* that were located in different clades. With a few exceptions, there was good correspondence between the MerA phylogeny and rhizobial systematics, where species of the same genus clustered together.

## Discussion

In this study, we analyzed the tolerance to Hg of several rhizobial strains collected from Hg-contaminated soils, and evaluated the role of mercuric reductase activity in the mercury tolerance of free-living bacteria and in the maintenance of nitrogen fixation in legume nodules subjected to mercury stress. The MIC values of all the strains used in this study were carefully re-evaluated, as in some preliminary mercuric reductase assays, some strains, which were initially considered as non-tolerant, displayed substantial mercuric reductase activity. The MIC values determined here for most *E. medicae* strains were similar to, although slightly higher than those reported in a previous study (Nonnoi et al., 2012), with a few of them that were considerably higher than those described earlier by Nonnoi et al. (2012). These differences could be attributed to modifications that might have occurred during the sub-culturing processes, in which inactive *merA* genes in the freshly collected strains might have been activated. The MIC values for *R. leguminosarum* were nearly identical to those reported previously (Nonnoi et al., 2012). The MIC values that we obtained for the *B. canariense* strains were consistently higher than those published earlier (Ruiz-Díez et al., 2012, Ruiz-Díez et al., 2013). The previously published MIC values for the *B. canariense* strains were determined in liquid medium, while in the present work, solid medium was used. The observed differences could be related to the fact that bacteria have the ability to form biofilms when they grow attached to a surface. Such biofilms are formed mainly by exopolysaccharides (EPS). The EPS matrix works as a diffusion barrier that slows down penetration of compounds such as toxic agents, antibiotics, and metals. Thus, the EPS matrix on the agar plates could be the cause for the observed higher tolerance to mercury. On the contrary, when bacteria are grown in suspension in liquid medium, they are dispersed and are more sensitive to toxic substances (Mah and O’Toole, 2001; Teitzel and Parsek, 2003). The presence of sensitive and tolerant strains in contaminated soils is common and might be due to several factors, such as the presence of micro-niches in the soil (Nonnoi et al., 2012).

A comparative promoter analysis could help determine a mechanism to explain the differences in mercuric reductase activity between strains, but this will require new genome assemblies and genome wide expression of sensitive and tolerant strains, data that are not available at the moment. Additionally, cis-regulatory differences could be also likely to explain differences between tolerant and sensitive strains; this would require experimental evidence rather than simple sequence comparisons. We are actually planning to test these hypotheses. Nevertheless, we feel that we have made a convincing case showing that the more tolerant *E. medicae* strains had significantly higher expression than non-tolerant strains, which is what we set out to do.

To our knowledge, there are no reports on the mechanisms that might account for mercury tolerance in rhizobia. Regarding the *mer* operon, variations in structure and organization have been found in different bacteria (Boyd and Barkay, 2012; Naguib et al., 2018). No generic *mer* operons have been identified up to now in the sequenced rhizobia genomes found in databases. Only *merA* and *merR* homologs have been identified in some of these genomes. The presence of *merA*, in the absence of any transporter (*merT*) and/or regulatory genes (*merR*), does not necessary imply effective Hg^2+^ reduction and mercury tolerance, as it has been observed in some bacteria lacking *mer* transporter genes (Osborn et al., 1997).

The mercuric reductase assays showed that the highly tolerant *E. medicae* strains presented the highest activities. We found a strong correlation between MIC values and mercuric reductase activity for this species. Others authors have also observed mercury tolerance involving mercury reductase activity in other types of bacteria lacking *mer* specific transporter genes, and it was postulated that protein transporters not specific to the *mer* system could be involved in mercury transport (Wang et al., 2009). In contrast, no correlation between MIC values and mercuric reductase activity was found for the *B. canariense* strains. This lack of correlation suggests that *B. canariense* might have other mercury tolerance mechanisms, such as the exopolysaccharide production mentioned above. In fact, the colonies formed by *B. canariense* strains appeared clearly more viscous or gelatinous than those formed by *E. medicae* or *R. leguminosarum*. This Hg-binding EPS mechanism most likely presents some intra-species variation, as the composition of the EPS matrix might vary, resulting in different MIC values among *B. canariense* strains. Due to the limited number of tolerant strains, the data for *R. leguminosarum* appear insufficient to determine whether there was a correlation between tolerance and mercuric reductase activity. In fact, it cannot be ruled out that different additional mechanisms might contribute to Hg tolerance in any of the three species.

When bacteria were grown in the presence of a sub-lethal Hg concentration, an increase in mercuric reductase activity could be observed for some strains. Most bacterial mercury detoxification systems are related to an induction phenomenon (Wang et al., 2009), and it is known that the expression of the *merA* gene, which encodes the MerA enzyme, can be induced by the presence of mercury (Freedman et al., 2012). Generic *mer* operons might carry regulatory genes. MerR is a transcriptional repressor which attaches to the promotor-operator region of the operon and acts as repressor or activator of the operon in the absence and presence of Hg^2+^, respectively (Barkay et al., 2003; Boyd and Barkay, 2012). We did not identify *merR* homologs in *E. medicae* or *R. leguminosarum* sequenced strains. Nonetheless, our results show a correspondence between mercuric reductase activity and *merA* expression, as both increased when bacteria were grown in the presence of a low Hg concentration, and *merA* expression in tolerant strains was higher than in sensitive strains. We analyzed *merA* expression in two *E. medicae* strains and two *R leguminosarum* strains with different Hg tolerance, and the results were similar for both species, suggesting the existence of a Hg-dependent regulation of *merA* expression and MerA activity. However, we cannot exclude that other strains might display different behavior.

It is known that when the differentiation from bacteria to bacteroids happens, there are physiological and genetic changes (Ampe et al., 2003). Some genes are induced while others are repressed or fully inactivated in the bacteroid. Here we showed that *merA* genes were expressed inside *M. truncatula* nitrogen-fixing nodules and the MerA was active. The expression patterns differed from those observed in free-living bacteria. Besides the clear differences between both experiments, the changes in genetic regulation that occur in the differentiation process could also explain the differences in *merA* gene transcription between the free-living *E. medicae* bacteria and bacteroids.

The expression of *merA1* and *merA* 2 in the different zones of the nodule was analyzed using the data of Roux et al. (2014) for expression of *Ensifer meliloti* genes in sections of *M. truncatula* nodules. *merA1* presented very low expression in the uninfected meristem. Expression in the FII zone, where the number of bacteria should be low, was high and similar to that of the infected zone FIII. This could indicate that *merA1* expression is lower in bacteroids than in undifferenciated bacteria, or could be due to some contamination of FII sections with infected cells. *merA2* displayed low expression in uninfected tissues (FI and FIId), and high expression in the fixation zone FIII.

Tolerant rhizobia strains constitute a promising bioremediation tool as they can survive in the presence of high metal concentrations (Hao et al., 2014). According to our results, a tolerant strain appears to be more efficient in reducing mercury on account of a higher basal *merA* expression. We and other groups have previously described that metal-tolerant rhizobia help maintain nitrogen fixation under metal stress conditions (Shvaleva et al., 2010; Rangel et al., 2017). Quiñones et al. (2013) showed in a long-term experiment that *Lupinus albus* plants grown in the presence of Hg and inoculated with a Hg-tolerant strain were more tolerant than those inoculated with a sensitive strain. This was likely due to the preservation of nitrogen fixation under mercury stress, but additional factors were involved. When the lupin plants were inoculated with a Hg-sensitive strain, nodulation was severely affected by mercury and a drastic drop in nitrogenase activity was observed. However, in their experimental conditions it was difficult to determine whether mercury was affecting nitrogen fixation itself, or the decrease in activity was a consequence of poor nodulation or formation of non-effective nodules. For these reasons, we designed a short-term experiment to affect nitrogen fixation alone, to show that the tolerance of the strain was important to maintain nitrogenase activity under mercury stress conditions. The present results suggest that nitrogen fixation is differentially affected by mercury stress depending on the mercury tolerance of the inoculated rhizobia.

The Hg-tolerant *E. medicae* strain used in this work and its protective effect on nitrogen fixation in the presence of Hg stress suggest a potential use as a bioremediation tool in Hg-contaminated environments. This could be exploited using the bacteria alone, due to their high mercuric reductase activity, or in association with tolerant *M. truncatula* varieties. In fact, we have analyzed *M. truncatula* germplasm for Hg tolerance (García de la Torre et al., 2013) and identified some Hg-tolerant cultivars. Moreover, *M. truncatula* is a forage legume that produces high biomass and has a good soil coverage. As a result, we consider that the combination of tolerant *E. medicae* strains and tolerant *M. truncatula* varieties has the potential to become a powerful tool for detoxification of Hg-contaminated soils.

## Data Availability Statement

The original contributions presented in the study are included in the article/Supplementary Material, further inquiries can be directed to the corresponding author.

## Author Contributions

TC, MML, and JJP contributed conception and design of the study. GA and PH determined the MIC values and the mercuric reductase activity of free living bacteria. TC, BP, GA, and PH designed primers and performed the qPCR assays. VL-D carried out the nitrogenase activity assays. DG-R and HPV determined the mercuric reductase activity in nodules. PT and DB contributed to the setup of the mercuric reductase assay. TP and TC contributed to the phylogeny analyses and to the discussion section of the manuscript. GA and DG-R wrote the first draft of the manuscript. JJP supervised and contributed to all the experiments in the manuscript. All authors contributed to manuscript revision, read, and approved the submitted version.

## Conflict of Interest

The authors declare that the research was conducted in the absence of any commercial or financial relationships that could be construed as a potential conflict of interest.
