# Supplementary material for: Mercury-Tolerant Ensifer medicae Strains Display High Mercuric Reductase Activity and a Protective Effect on Nitrogen Fixation in Medicago truncatula Nodules Under Mercury Stress
Source: Front Plant Sci. 2021 Jan 14;11:560768. doi: 10.3389/fpls.2020.560768 (PMC7840509; doi:10.3389/fpls.2020.560768)
Supplement: Supplementary file 1 [file Data_Sheet_1.PDF]

(A)

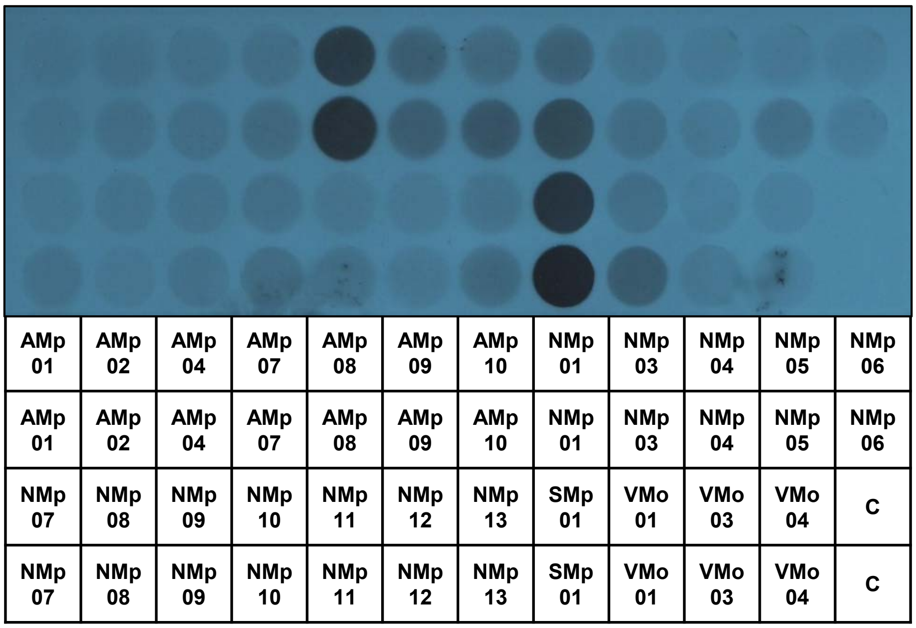

1	AMp 01	AMp 02	AMp 04	AMp 07	AMp 08	AMp 09	AMp 10	NMp 01	NMp 03	NMp 04	NMp 05	NMp 06
2	AMp 01	AMp 02	AMp 04	AMp 07	AMp 08	AMp 09	AMp 10	NMp 01	NMp 03	NMp 04	NMp 05	NMp 06
3	NMp 07	NMp 08	NMp 09	NMp 10	NMp 11	NMp 12	NMp 13	SMp 01	VMo 01	VMo 03	VMo 04	C
4	NMp 07	NMp 08	NMp 09	NMp 10	NMp 11	NMp 12	NMp 13	SMp 01	VMo 01	VMo 03	VMo 04	C

(B)

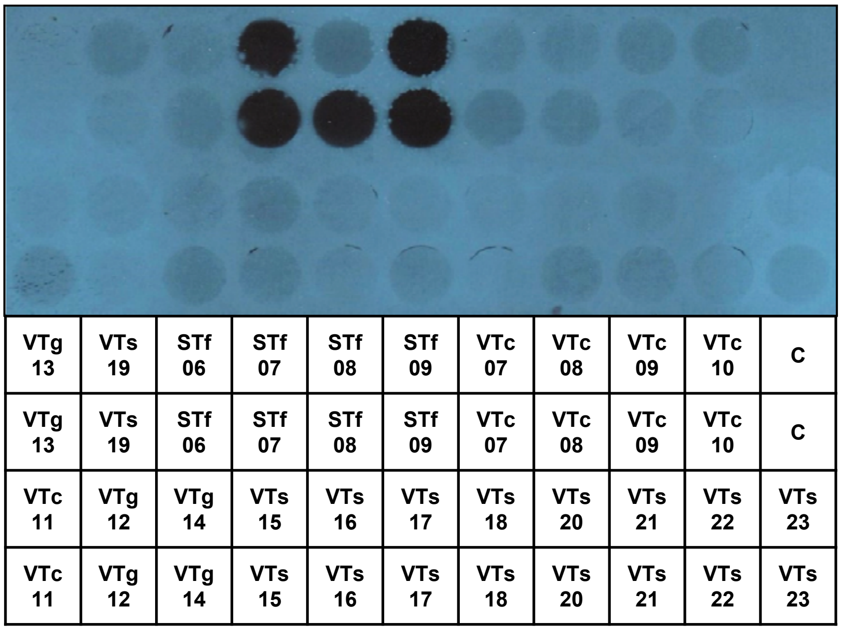

1	VTg 13	VTs 19	STf 06	STf 07	STf 08	STf 09	VTc 07	VTc 08	VTc 09	VTc 10	C
2	VTg 13	VTs 19	STf 06	STf 07	STf 08	STf 09	VTc 07	VTc 08	VTc 09	VTc 10	C
3	VTc 11	VTg 12	VTg 14	VTs 15	VTs 16	VTs 17	VTs 18	VTs 20	VTs 21	VTs 22	VTs 23
4	VTc 11	VTg 12	VTg 14	VTs 15	VTs 16	VTs 17	VTs 18	VTs 20	VTs 21	VTs 22	VTs 23

(C)

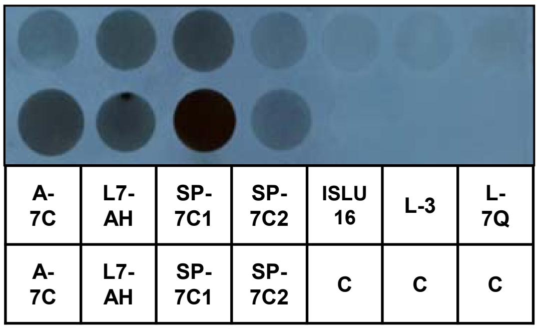

1	A-7C	L7-AH	SP-7C1	SP-7C2	ISLU 16	L-3	L-7Q
2	A-7C	L7-AH	SP-7C1	SP-7C2	C	C	C

Supplementary Figure 1. Mercuric reductase activity of rhizobial strains. (A) *Ensifer medicae*. (B) *Rhizobium leguminosarum* bv. *trifolii*. (C) *Bradyrhizobium canariense*. The darkened areas on the X-ray films were the result of the reduction of Ag^{+} emulsion by mercury vapors produced by the bacterial reduction of Hg2+ to Hg^{0}, which is the result of the mercuric reductase activity of the strains. Below the X-ray films the bacterial strains are indicated. C: Control. Rows 1 and 3 show the activity of the strains grown in the absence of $HgCl_2$. Rows 2 and 4 show the activity of the strains grown in the presence of 4 µM $HgCl_2$, showing that activity is inducible in some strains. To quantify activity, a densitometry analysis of the dark spots was performed.
